# Development, validation, and visualization of a web-based nomogram to predict the effect of tubular microdiscectomy for lumbar disc herniation

**DOI:** 10.3389/fsurg.2023.1024302

**Published:** 2023-03-20

**Authors:** Xinyao Chen, Fabin Lin, Xiongjie Xu, Chunmei Chen, Rui Wang

**Affiliations:** Department of Neurosurgery, Pingtan Comprehensive Experimental Zone Hospital, Union Hospital, Fujian Medical University, Fuzhou, China

**Keywords:** lumbar disc herniation, tubular microdiscectomy, nomgram, web calculator, prediction mode

## Abstract

**Objective:**

The purpose of this study was to retrospectively collect the relevant clinical data of lumbar disc herniation (LDH) patients treated with the tubular microdiscectomy (TMD) technique, and to develop and validate a prediction model for predicting the treatment improvement rate of TMD in LDH patients at 1 year after surgery.

**Methods:**

Relevant clinical data of LDH patients treated with the TMD technology were retrospectively collected. The follow-up period was 1 year after surgery. A total of 43 possible predictors were included, and the treatment improvement rate of the Japanese Orthopedic Association (JOA) score of the lumbar spine at 1 year after TMD was used as an outcome measure. The least absolute shrinkage and selection operator (LASSO) method was used to screen out the most important predictors affecting the outcome indicators. In addition, logistic regression was used to construct the model, and a nomogram of the prediction model was drawn.

**Results:**

A total of 273 patients with LDH were included in this study. Age, occupational factors, osteoporosis, Pfirrmann classification of intervertebral disc degeneration, and preoperative Oswestry Disability Index (ODI) were screened out from the 43 possible predictors based on LASSO regression. A total of 5 predictors were included while drawing a nomogram of the model. The area under the ROC curve (AUC) value of the model was 0.795.

**Conclusions:**

In this study, we successfully developed a good clinical prediction model that can predict the effect of TMD for LDH. A web calculator was designed on the basis of the model (https://fabinlin.shinyapps.io/DynNomapp/).

## 1. Introduction

Lumbar disc herniation (LDH) is a degenerative disease of the lumbar spine and a common cause of low back and leg pain (1, 2). Lumbar intervertebral disc degeneration is a fundamental factor in the pathogenesis of LDH. At the same time, poor use of the waist, such as prolonged sitting, squatting, or bending over weight-bearing for a long time, will cause cumulative damage to the lumbar intervertebral disc, resulting in rupture of the lumbar intervertebral disc and nucleus pulposus (3). Some studies have shown that genetic factors, obesity, and diabetes may also be the potential causes of LDH (3, 4). The protruding intervertebral disc tissue stimulates or compresses the nerve root and cauda equina, causing a series of signs and symptoms, such as lumbar and leg pain, cauda equina syndrome symptoms, sensory disturbance, and decreased muscle strength. Lumbar discectomy is the main surgical method for the treatment of LDH. The purpose of the operation is to relieve the irritation or compression caused by the herniated intervertebral disc tissue, thereby relieving the patient's pain and improving the symptoms of nerve damage (5).

Our previous meta-analysis has shown that compared with traditional posterior open surgery, tubular microdiscectomy (TMD) technology for the treatment of lumbar intervertebral disc disease is beneficial for shortening the operation time, reducing postoperative pain, shortening the hospitalization period, and reducing the risk of peripheral nerve fibrosis and spinal instability (6).

A clinical prediction model is a mathematical model that uses a multi-factor model to estimate the probability that a subject is currently suffering from a disease or a certain outcome in the future (7–9). Depending on the clinical question of the study, clinical prediction models include diagnostic models, prognostic models, and disease occurrence models for predicting the occurrence of a disease.

Studies have shown that patients are more satisfied with LDH surgery if their preoperative expectations regarding the clinical outcomes are met. Therefore, surgeons need reliable predictive tools to provide patients with personalized estimates of probabilities associated with clinical outcomes, thereby helping them form realistic clinical expectations before surgery, better understand the future associated risks, and guide them and the physicians to collectively decide whether to proceed with further treatment when possible adverse events arise (9). Currently, many clinical prediction models have been developed to predict the prognosis of spinal surgery, but there are no relevant research reports at home and abroad on the development and validation of relevant clinical prognosis models for TMD in the treatment of patients with LDH.

The purpose of this study was to retrospectively collect the relevant clinical data of patients with LDH who received TMD technology to establish a prognostic model to predict the treatment improvement rate in LDH patients at 1 year after TMD, and then to evaluate the performance of the prognostic model and clinical outcomes. The efficacy was validated and evaluated, and the prognostic model was finally used in clinical practice.

## 2. Methods

### 2.1. Patient collection

The clinical study was approved by the Ethics Committee of Fujian Medical University Union Hospital on February 15, 2022, with the ethics approval number: 2022KY026. From January 2018 to January 2021, a total of 273 patients, who were sent to the department of neurosurgery, Fujian Medical University Union Hospital owing to LDH and received TMD treatment, were enrolled in this research.

The selection criteria were as follows: (1) Age of selection: 12–85 years; (2) Typical sciatica with or without low back pain and other symptoms; (3) Standard conservative treatment was ineffective for more than 3 months and seriously affected their life, or was accompanied by severe pain, cauda equina dysfunction, decreased muscle strength, muscle atrophy, and other symptoms; (4) The straight leg raising test on the affected side was less than or equal to 70°; (5) Herniation of the lumbar intervertebral disc was confirmed by CT and MRI and the position of the patient was consistent with the corresponding neurological symptoms; (6) receipt of TMD technology treatment.

The exclusion criteria were as follows: (1) those with missing imaging data or who could not be followed up as required; (2) frontal and lateral lumbar spine x-rays and x-rays in hyperextension and hyperflexion showed segmental lumbar instability; (3) in combination with other severe physical or mental illness; (4) patients with rheumatic autoimmune diseases that may cause similar symptoms; (5) those who were participating in other clinical trials.

All surgeries were performed by the neurospine surgeons of our institution.

(1) Patient position: prone position.
(2) Anesthesia method: endotracheal intubation, intravenous inhalation combined with general anesthesia.
(3) Preoperative positioning: After setting the body position, under the frontal and lateral perspective of the C-arm machine, the positioning needle was used to locate the surgical segment, and the skin over the surgical lumbar segment was accurately marked.
(4) Positioning puncture: After routine disinfection and draping of towels, the patient's surgical segment and puncture site were positioned and confirmed according to the intraoperative C-arm x-ray frontal and lateral fluoroscopic films, and pain symptoms in the lower limbs on the midline after the level of the surgical segment were detected. A longitudinal incision measuring about 1.5–1.8 cm in length was created by making a 1.5–2.0 cm lateral incision on the side (or the side with severe symptoms), followed by incision of the subcutaneous tissue and muscle fascia. A Kirschner wire was punctured from the incision to the lower edge of the medial lamina of the upper vertebral body of the operative segment, and the depth and angle of the Kirschner needle puncture were determined by C-arm fluoroscopy. After confirming that the Kirschner wire had reached the target point, the paravertebral dilation cannula was used to bluntly dilate and separate the paravertebral muscles step by step along the Kirschner wire. A surgical microchannel was placed along the dilation cannula and fixed, and all dilation cannulae were removed and repeated. x-ray positioning of the C-arm machine was performed to determine the alignment of the microchannel with the intervertebral disc space of the surgical segment.
(5) Laminar shaping: After bluntly separating the soft tissue on the surface of the lamina in the microchannel field of view under the microscope, a high-speed grinding drill removed the lower edge of the lamina and the bone near the lower edge of the spinous process of the upper lumbar vertebra in the operating segment. The intraspinal ligamentum flavum was excised, the spinal canal was fully exposed, and the posterior aspect of the dura mater and the bilateral nerve root sleeves were completely exposed. Along the ipsilateral nerve root, the lateral ligamentum flavum was excised, and the inferior lamina was removed. On the upper edge, the nerve root was fully exposed from the point of leaving the dura mater to entering the lower vertebral canal; the ipsilateral nerve root was gently retracted to the midline to expose the protruding or prolapsed intervertebral disc, and the protruding or prolapsed nucleus pulposus tissue and part of the nucleus pulposus in the intervertebral space were removed. The nucleus pulposus in the space was decompressed in a 360° circular manner to the nerve root, and the spinal canal and neural foramen were re-explored.
(6) After complete hemostasis was achieved, the ipsilateral nerve root cuff was found to be pulsating well, and the spinal canal was fully decompressed outside the dural sac. The surgical microchannel was withdrawn, and the paravertebral muscles were repositioned. After careful hemostasis, the sarcolemma, subcutaneous tissue, and skin were sutured.

### 2.2. Data collection

In order to construct and verify the evaluation prediction model, this study retrospectively collected the relevant clinical data of patients with LDH who fulfilled the inclusion and exclusion criteria. The possible predictive factors included the 43 items presented below, and 1 year after TMD, the treatment improvement rate of the lumbar JOA score was used as an outcome measure.

#### 2.2.1. Basic information

Age, gender, height, weight, body mass index (BMI), high-risk occupation (an occupation that requires long-term sedentary standing or heavy physical activity), and family history (a first-degree relative with LDH).

#### 2.2.2. Medical history examination

History of lumbar trauma, duration of disease, preoperative conservative treatment time, preoperative pain medication use time, low back pain, underlying diseases (hypertension and diabetes), smoking history, alcoholism, preoperative physical examination (straight leg raising test) angle, sensory disturbance, muscle strength grading of the affected limb, and Barthel's scale.

#### 2.2.3. Preoperative inspection indicators

Serum creatine kinase (CK) and serum albumin (ALB).

#### 2.2.4. Preoperative examination indicators

**Figure 1:**
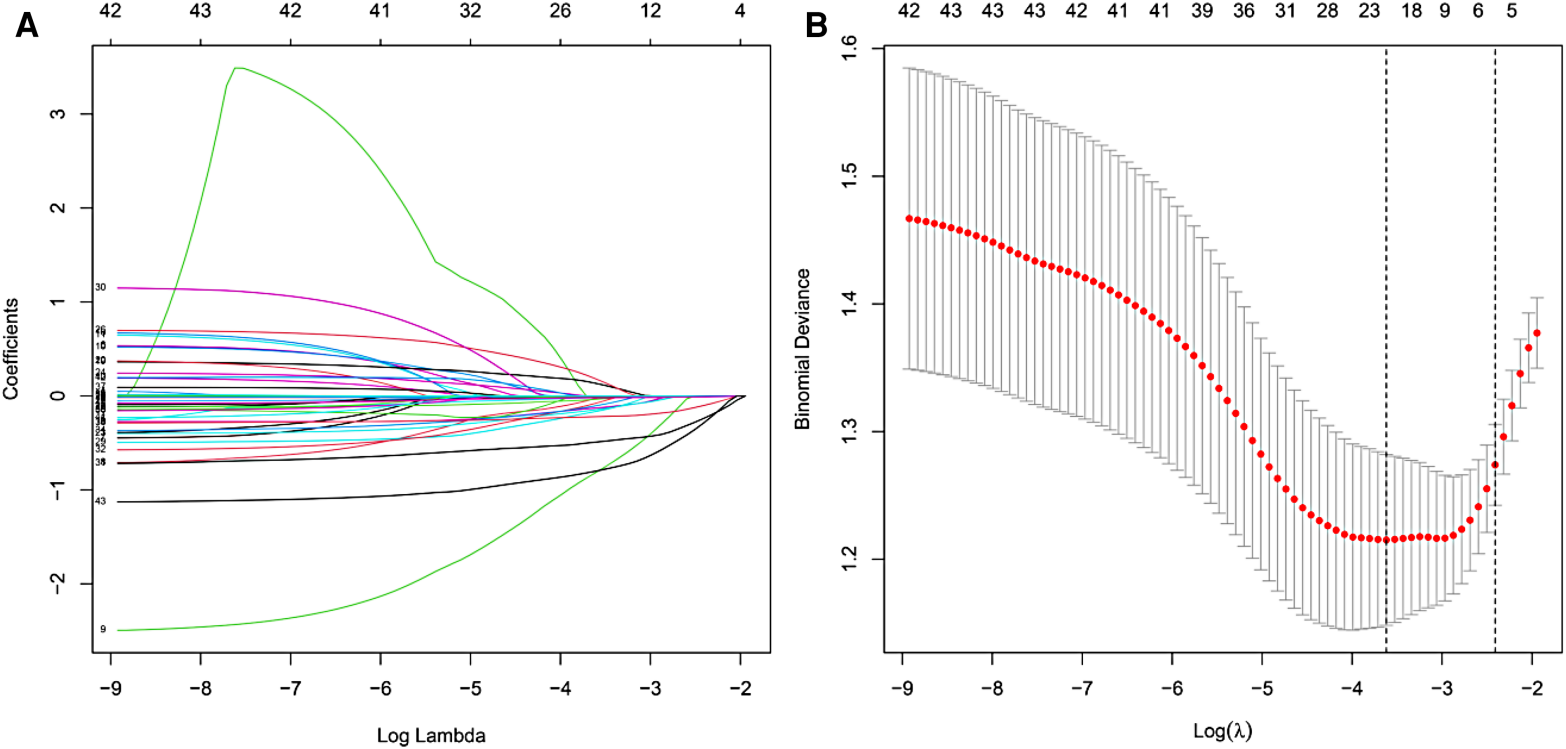
LASSO coefficient profiles at the optimal lambda value selected using 10-fold cross-validation (**A**) Optimal parameter (lambda) selection in the LASSO model using 10-fold cross-validation *via* minimum criteria (**B**).

(1) Degeneration of the lumbar spine: ligament calcification, osteoporosis, lumbar spondylolisthesis, lumbar intervertebral disc space collapse, lumbar spinal canal sagittal meridian, and Modic type (10) changes in the endplate and subendplate (Figure 1).
(2) Related conditions of lumbar intervertebral disc herniation: the number of protruding segments, the protruding position of the responsible segment, the sagittal division of the responsible segment (11), transverse division of the responsible segment and the degree of herniation according to the Michigan State University (MSU, MSU) classification grade (12), and Pfirrmann grade (13) for lumbar intervertebral disc degeneration of the responsible segment.

#### 2.2.5. Preoperative scoring

(1) The American Society of Anesthesiologists (ASA) score (14): before anesthesia, the patient was divided into 5 grades according to the patient's physical condition and surgical risk (grade 1 means no other systemic diseases except local lesions, grade 2 indicates mild or moderate systemic disease, grade 3 indicates that the patient has any serious systemic disease but has not lost the work ability, grade 4 indicates that the patient has a serious life-threatening systemic disease and has lost the work ability, and grade 5 indicates that the patient is in a critical condition requiring emergency surgery).
(2) Oswestry Disability Index (ODI) score (15, 16): a total score of 10 for the pain level, self-care ability of daily living, carrying objects, walking, sitting, standing, sleep, sex life, social activities, and travel was assessed by a questionnaire. Each aspect has 1 question and 6 options, corresponding to 0 to 5 points, respectively. The ODI score was calculated as the percentage of the total score obtained by the subjects in the total score of the highest score of the evaluated item. The higher the score, the more severe the dysfunction.
(3) Numerical rating scale (NRS) score (17) for preoperative low back pain and leg pain: a score of 0–10 indicates the degree of pain; the higher the value, the higher the degree of pain.
(4) The Japanese Orthopedic Association (JOA) evaluation treatment score (18) for the lumbar spine before surgery: including subjective symptoms (such as the degree of low back pain, numbness or pain in the lower extremity, and walking ability), clinical signs (such as straight leg lift) High angle, sensory disturbance, muscle strength 3 items), the degree of limitation of daily activities (including bed rest, standing, washing, bending, sitting, lifting, and walking; 7 items) and bladder function in a total of 4 aspects, each with 3-. The four choices correspond to their respective scores, with a total score (18). The lower the score, the more obvious the dysfunction.

#### 2.2.6. Surgery-related indicators

Surgical segments, number of operative segments, operative time, and intraoperative blood loss were considered as the surgery-related indicators.

#### 2.2.7. Outcome indicators

Treatment improvement rate score (18, 19) for the lumbar spine JOA score at 1 year after TMD: The lumbar spine JOA score method at 1 year after the operation was the same as that before the operation, and the treatment improvement rate was =[(post-treatment score—pre-treatment score) ÷ (full score 29—pre-treatment score)] × 100%. The improvement rate reflects the improvement of the lumbar spine function of the patient before and after treatment to evaluate the clinical efficacy: a cure rate of 100% was considered as cure, an improvement rate of more than 60% was considered as markedly effective, 25%–60% was considered as effective, and less than 25% was considered invalid. Patients with an improvement rate of the lumbar spine JOA score >60% (a significant curative effect or cure) at 1 year after TMD were assigned to group A, and patients with an improvement rate of the lumbar spine JOA score ≤60% (effective but insignificant or ineffective) were assigned to group B.

### 2.3. Statistical analysis

#### 2.3.1. Variable screening

All the included predictors were treated as continuous or categorical variables, and the outcome indicators were treated as dichotomous variables to organize the data set. Continuous variables were represented by mean and standard deviation, and categorical variables were represented by frequency and percentage. The least absolute shrinkage and selection operator (LASSO) method was used to reduce the number of variables and the possibility of model overfitting, and it was combined with the clinical experience to screen out the most effective rate of lumbar JOA improvement at 1 year after the operation.

#### 2.3.2. Model construction

For the selected predictors and outcome indicators, a binary logistic regression method was used to build a model, and a nomogram of the prediction model was drawn to visualize the abstract results of logistic regression.

#### 2.3.3. Model verification and evaluation

In the original data set, the bootstrap resampling method was used for internal verification of the model, and the area under the curve (AUC) was calculated by the receiver operating characteristic (ROC) curve to verify the discrimination of the model. A calibration curve was used to verify the calibration of the model. Finally, decision curve analysis (DCA) was used to evaluate the clinical utility of the model. All data analyses were performed using R software (version 4.1.2).

## 3. Results

### 3.1. Clinical data

A total of 273 patients with lumbar intervertebral disc herniation were included in this study. Among them, the treatment improvement rate of the lumbar spine JOA score at 1 year after TMD was more than 60% (a significant curative effect or cured); it accounted for 57.1% of patients (156 cases) who were classified as group A, and the treatment improvement rate of the lumbar spine JOA score at 1 year after TMD was less than or equal to 60% (effective but not significant or ineffective); it accounted for 42.9% of patients (117 cases) who were classified as group B. The average age of patients in group A was 48.51 ± 13.87 years, the proportion of males was 52.6% (82 cases), and the proportion of patients with postoperative JOA score improvement rate equal to 100% (i.e., complete cure) was 7.7% (12 cases) in group A. The average age of patients in group B was 56.49 ± 14.18 years, the proportion of males was 54.7% (64 cases), and the proportion of patients with postoperative JOA score improvement rate less than 25% (i.e., invalid) was 12.0% (14 cases) in group B. The basic data, relevant medical history, preoperative examination, test and related scores, surgery-related data, and other clinical data of the two groups of patients were described by continuous or categorical variables, as shown in Table 1.

**Table 1:** Baseline data of the training group and the validation group.

Predictor	A group *N* (%) or Mean ± SD	B group *N* (%) or Mean ± SD
Age/year	48.51 ± 13.87	56.49 ± 14.18
**Gender**		
Male	82 (52.6%)	64 (54.7%)
Female	74 (47.4%)	53 (45.3%)
Height/m	1.65 ± 0.09	1.63 ± 0.07
Weight/kg	64.53 ± 10.59	66.21 ± 12.45
BMI/kg/m^2^	24.69 ± 3.54	24.69 ± 3.54
**High risk occupation**		
Yes	55 (35.3%)	73 (62.4%)
No	101 (64.7%)	44 (37.6%)
**Family history**		
Yes	26 (16.4%)	25 (21.4%)
No	130 (83.3%)	92 (78.6%)
**Medical history examination**		
**History of lower back trauma**		
Yes	7 (4.5%)	4 (3.4%)
No	149 (95.5%)	113 (96.6%)
**Disease duration**		
<6 mounths	71 (45.5%)	48 (41.0%)
≥ 6 mounths, <12 mounths	22 (14.1%)	18 (15.4%)
≥12 mounths, <24 mounths	20 (12.8%)	12 (10.3%)
≥24 mounths	43 (27.6%)	39 (33.3%)
**Conservative treatment time**		
<3 mounths	73 (46.8%)	46 (39.3%)
≥3 mounths, <6 mounths	21 (13.5%)	25 (21.4%)
≥6 mounths	62 (39.7%)	46 (39.3%)
**Preoperative use of analgesics**		
Yes	52 (33.3%)	53 (45.3%)
No	104 (66.7%)	64 (54.7%)
**Low back pain**		
Yes	109 (69.9%)	92 (78.6%)
No	47 (30.1%)	25 (21.4%)
**High blood pressure**		
Yes	27 (17.3%)	35 (30.0%)
No	129 (82.7%)	82 (70.0%)
**Diabetes**		
Yes	10 (6.4%)	18 (15.4%)
No	146 (93.6%)	99 (84.6%)
**Smoking history**		
Yes	36 (23.1%)	29 (24.8%)
No	120 (76.9%)	88 (75.2%)
**History of alcoholism**		
Yes	5 (3.2%)	15 (12.8%)
No	151 (96.8%)	102 (87.2%)
**Straight leg raise test angle**		
<40°	46 (29.5%)	27 (23.1%)
≥40°, <60°	65 (41.7%)	53 (45.3%)
≥60°, ≤70°	45 (28.8%)	37 (31.6%)
**Degree of sensory impairment**		
No	91 (58.3%)	53 (45.3%)
Slight	57 (36.5%)	46 (39.3%)
Obvious	8 (5.1%)	18 (15.4%)
**Affected limb muscle strength classification**		
<4 level	1 (0.6%)	4 (3.4%)
4 level	34 (21.8%)	34 (29.1%)
5 level	120 (76.9%)	79 (67.5%)
**Preoperative Barth's sign**		
Negative	153 (98.1%)	112 (94.0%)
Positive	3 (1.9%)	7 (6.0%)
**Inspection index**		
Serum creatine kinase/U/L	100.89 ± 67.34	103.27 ± 121.17
Serum albumin/g/L	41.91 ± 3.99	41.30 ± 3.71
**Check metrics**		
**Ligament calcification**		
Yes	74 (47.4%)	73 (62.4%)
No	82 (52.6%)	44 (37.6%)
Osteoporosis		
Yes	12 (7.7%)	34 (29.1%)
No	144 (92.3%)	83 (70.9%)
**Spondylolisthesis**		
Yes	8 (5.1%)	12 (10.3%)
No	148 (94.9%)	105 (89.7%)
**Intervertebral space collapse**		
Yes	35 (22.4%)	43 (36.8%)
No	121 (77.6%)	74 (63.2%)
Sagittal meridian of lumbar spinal canal/cm	1.46 ± 0.24	1.51 ± 0.25
**Modic**		
Normal	66 (42.3%)	39 (33.3%)
I type	25 (16.0%)	22 (18.8%)
II type	29 (18.6%)	26 (22.2%)
III type	36 (23.1%)	30 (25.7%)
**Number of prominent segments**		
1 segment	85 (54.5%)	44 (37.6%)
2 segments	50 (32.1%)	45 (38.4%)
3 segments	15 (9.6%)	14 (11.9%)
4 segments	4 (2.6%)	12 (10.9%)
5 segments	2 (1.3%)	2 (1.7%)
**Responsibility segment prominent orientation**		
Left	79 (50.6%)	70 (59.8%)
Right	77 (49.4%)	47 (40.2%)
**Sagittal division of responsible segment**		
Zone 2	2 (1.3%)	1 (0.9%)
Zone 1	8 (5.1%)	5 (4.3%)
Zone 0	58 (37.2%)	59 (50.4%)
Zone −1	72(46.2%)	44(37.6%)
Zone −2	16 (10.2%)	8 (6.8%)
**Responsible segment transection zoning**		
Zone A	65 (41.7%)	52 (44.4%)
Zone B	61 (39.1%)	47 (40.2%)
Zone C	30 (19.2%)	18 (15.4%)
**Grading of the prominence degree of the cross section of the responsible segment**		
1 level	60 (38.5%)	41 (35.0%)
2 level	65 (41.7%)	47 (40.2%)
3 level	31 (19.8%)	29 (24.8%)
**Pfirrmann classification of responsible segmental disc degeneration**		
2 level	21 (13.5%)	10 (8.5%)
3 level	70 (44.9%)	32 (27.4%)
4 level	52 (33.3%)	37 (31.6%)
5 level	13 (8.3%)	38 (32.5%)
**Preoperative scoring**		
**ASA score**		
1 level	122 (78.2%)	73 (62.4%)
2 level	33 (21.2%)	37 (31.6%)
3 level	1 (0.6%)	7 (6.0%)
ODI score /%	49.72 ± 15.08	59.88 ± 18.02
Low back pain NRS	2.61 ± 2.01	3.52 ± 2.05
Leg pain NRS	4.87 ± 1.25	5.14 ± 1.29
Lumbar spine JOA score	13.53 ± 3.11	11.84 ± 3.63
**Surgical index**		
**Surgical segment/例**		
L1/2, L2/3, L3/4	9 (5.8%)	17 (14.5%)
L4/5	89 (56.4%)	65 (55.6%)
L5/S1	57 (36.5%)	27 (23.1%)
Multi-segment	2 (1.3%)	8 (6.8%)
**Surgical segment**		
Single segment	154 (98.7%)	109 (93.2%)
Multi-segment	2 (1.3%)	8 (6.8%)
Operation time/h	2.69 ± 0.86	2.98 ± 1.09
Intraoperative blood loss/ml	20.04 ± 10.36	25.86 ± 23.97

Table 1: Comparison of data between group A (postoperative improvement rate > 60%) and group B (postoperative improvement rate ≤ 60) (continued).

### 3.2. Variable filter

In the collected data set, LASSO regression was used to screen out the 5 most important predictors affecting the improvement rate of lumbar spine JOA score at 1 year after surgery, including age, high-risk occupation, osteoporosis, the Pfirrmann classification of intervertebral disc degeneration at the responsible segment, and the preoperative ODI score, from among the 43 possible predictors included (Figures 1,B).

### 3.3. Model building, and nomogram construction and verification

**Figure 2:**
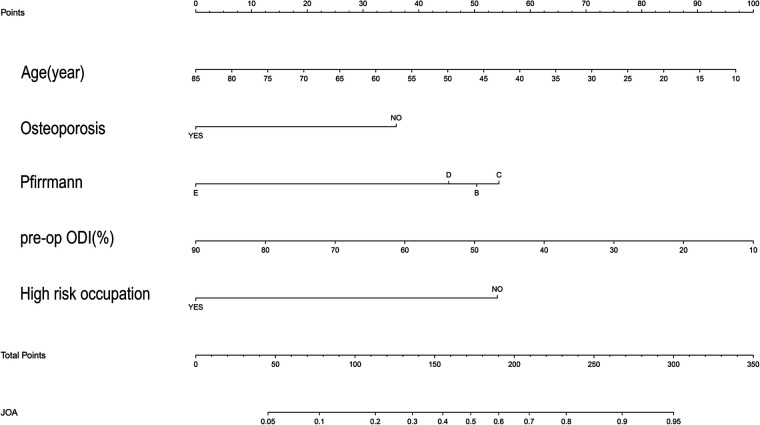
Nomogram for the risk of the improvement of JOA for patients with LDH.

After sorting out the 5 selected predictors and outcome indicator data sets, binary logistic regression was used to construct the model, and a nomogram of the model was drawn (Figure 2).

**Figure 3:**
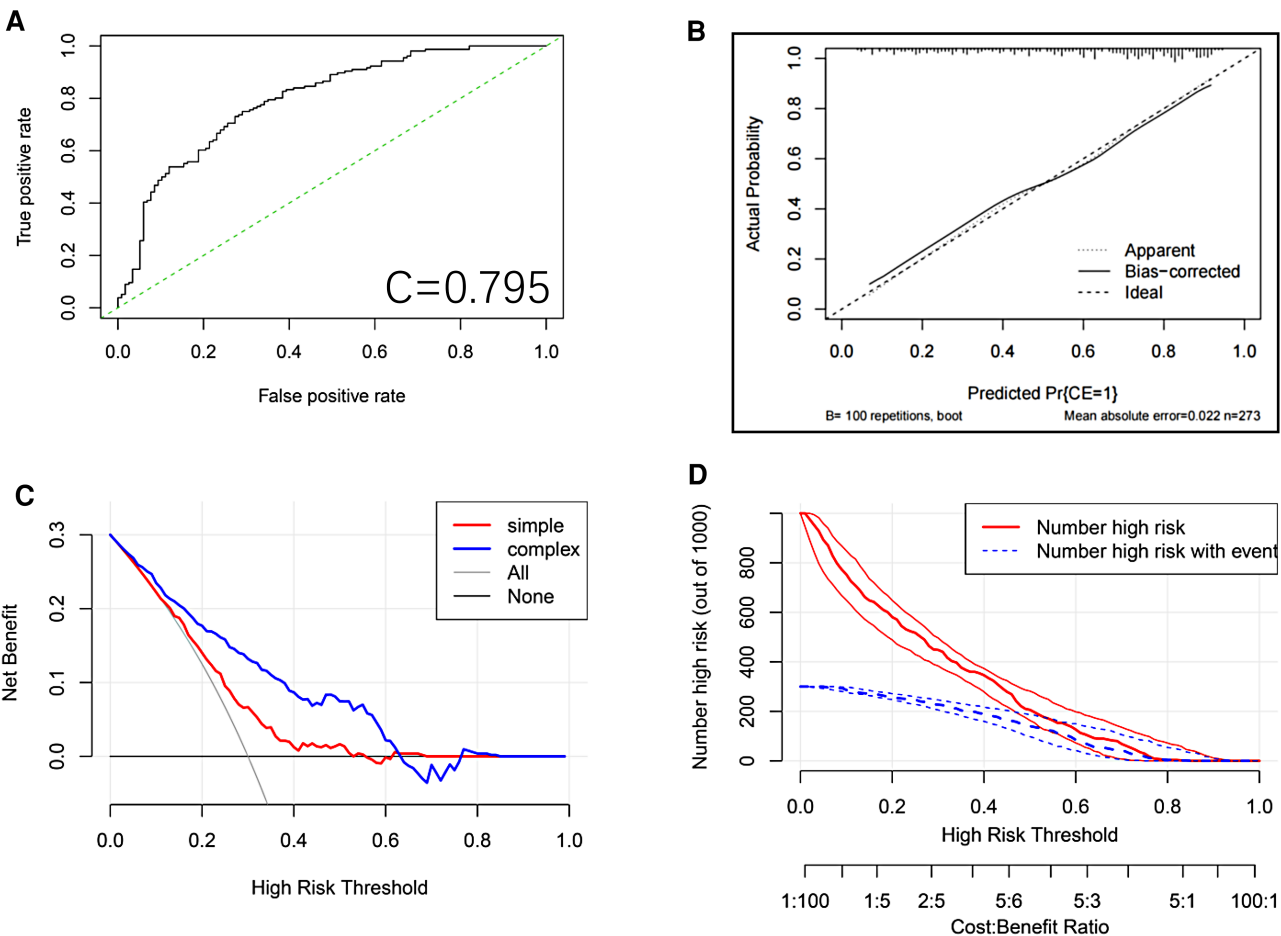
ROC curve of the logits regression model. (**A**) Calibration plot for the nomogram. The apparent and bias corrected values are close to each other, which means that the nomogram has good predictive performance. (**B**) Nomogram decision curve (DCA) for the risk of the lung metastasis. The red curve (number of high risk) indicates the number of people classified as positive (high risk) by the nomogram for each threshold probability. The green curve (number of high risk with the outcome) represents the number of true positive under each threshold probability. (**C**) Clinical impact analysis of the nomogram (**D**).

We plotted the ROC curve of the Logits regression model and calculated the AUC, which was found to be 0.795 (Figure 3A).

To validate the model internally, we performed 5X400 K-fold cross-validation, and calculated the AUC values, which were found to be Min 0.4376, Mean 0.7704, and Max. 0.9386.

After the model was constructed, the model was internally validated by using the bootstrap resampling method from the original data set. The calibration curve of the model (Figure 3B) showed that the linear regression slope between the probability predicted by the model and the actual situation was close to 1.

Finally, the DCA method was used to draw the DCA curve (Figure 3C) to evaluate the clinical utility of the model. The results showed that in most of the threshold ranges, the net benefit rate of the model was higher than that of the extreme curve. The clinical impact curve showed that a consistent advantage was predicted for high-risk patients within the most favorable threshold probability and acceptable cost-effectiveness (Figure 3D).

### 3.4. An online dynamic nomogram

**Figure 4:**
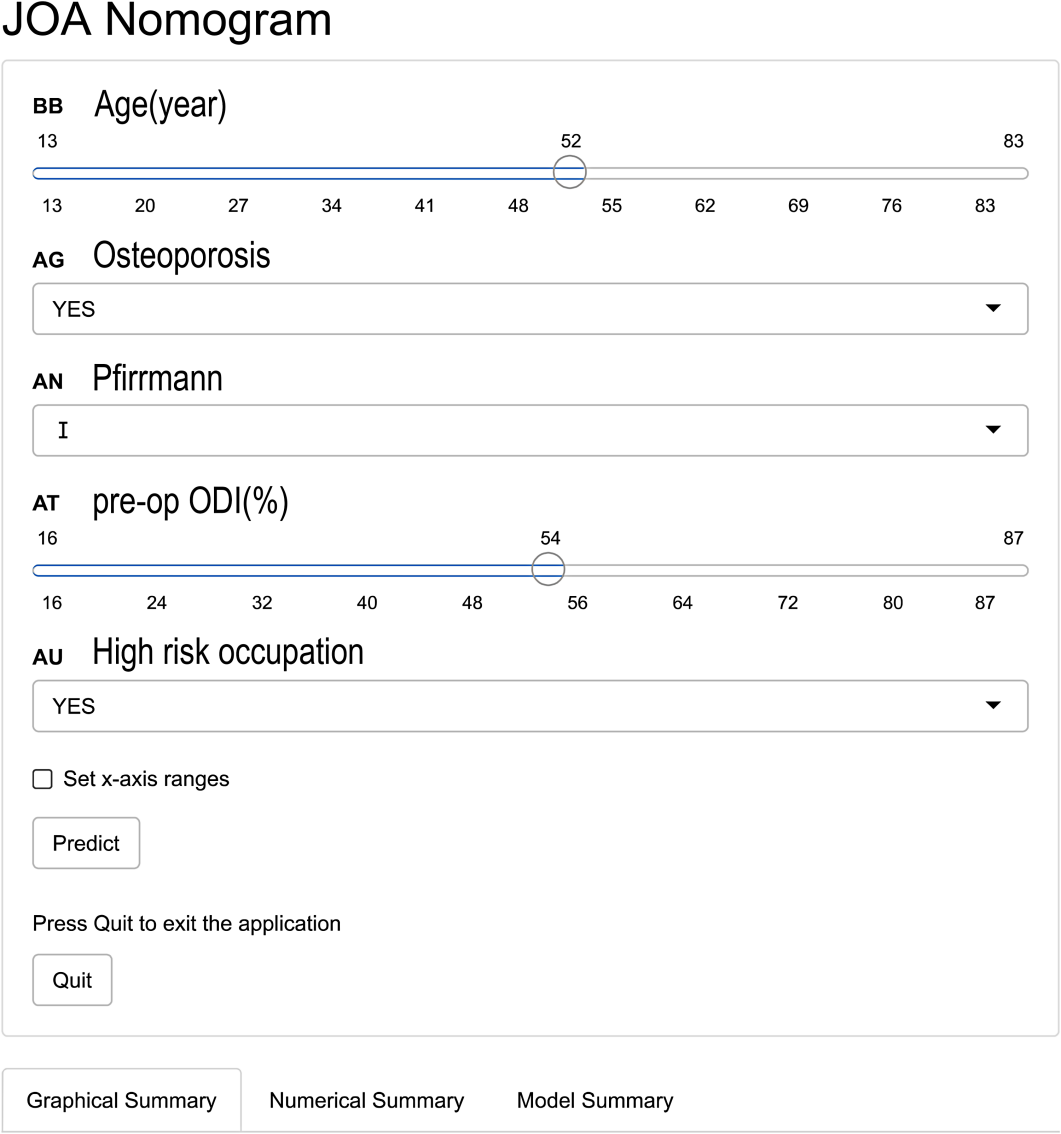
The web-based calculator for predicting the improvement of JOA in patients with LDH.

To conveniently predict the effect of TMD for LDH, we developed a dynamic nomogram on the website (https://fabinlin.shinyapps.io/DynNomapp/) (BB: Age(year), AG: Osteoporosis, AN: Pfirrmann, AT: pre-op ODI (%), AU: High risk occupation). By entering the specific information of lumbar disc herniation in the web-online tool, we could obtain the effect of TMD for LDH (Figure 4).

## 4. Discussion

This study developed a nomogram to predict the rate of improvement in the lumbar JOA score at 1 year after TMD. Based on the clinical experience and relevant literature reviewed, this study initially selected 43 possible predictors. Based on the LASSO results, we only selected 5 predictors, including age, occupational factors, osteoporosis, Pfirrmann classification of the responsible segmental disc degeneration, and preoperative ODI score. In this study, we provided a convenient nomogram prediction model and a web calculator on the basis of the model (https://fabinlin.shinyapps.io/DynNomapp/) (BB: Age(year), AG: Osteoporosis, AN: Pfirrmann, AT: pre-op ODI(%), AU: High risk occupation). In addition, studies have shown that gender, BMI, duration of disease, diabetes, smoking history, Modic changes, sagittal division, MSU grading, and other factors have a certain correlation with the postoperative efficacy for LDH (11, 19–22). However, in the present study, these factors were less correlated with the rate of improvement in the JOA score after TMD, which may be due to different treatment modalities or different sample populations. Based on the data set of the selected predictors, we constructed a nomogram of the model and used the bootstrap resampling method to internally validate the model through the original data set. The AUC calculated from the ROC curve of the model was 0.795, which indicated that the prediction model had good discriminating ability and could correctly distinguish the future population with different curative effects. The calibration curve of the model showed that the probability predicted by the model was in good agreement with the actual situation, indicating that the predicted risk of the model had a high degree of consistency with the actual risk, and the model had high accuracy. The DCA curve showed that the net benefit rate of the model was higher than that of the extreme curve in most of the threshold intervals, indicating that the clinical utility of the model was good (7, 8). To sum up, the prognostic model designed in this study had good discrimination, calibration, and clinical efficacy, and it only included 5 predictors, which made the operation simple and convenient, and could be widely used in daily clinical work for the treatment of patients undergoing TMD surgery.

### 4.1. Analysis of predictors

Although a number of studies have conducted multivariate analysis of the related risk factors affecting the postoperative efficacy for LDH, there are still few research reports on the related factors affecting the improvement rate of the lumbar JOA score after TMD. This study found that age, occupational factors, osteoporosis, Pfirrmann classification of intervertebral disc degeneration, and preoperative ODI score were important risk factors affecting the improvement rate of the lumbar JOA score after TMD.

The results of this study suggest that older patients may have worse clinical outcomes, which is consistent with the findings of most previous studies (23, 24). The reasons for this occurrence may be multifaceted. The results presented by Gruber et al. (25) showed that with increasing age, the healing process of the outer plate after surgery was not be enough to effectively reconstruct the outer ring, and the lower back muscles were atrophied in older patients than in younger patients and the muscle group's support for the lumbar spine was weakened, which was more likely to cause postoperative recurrence of intervertebral disc herniation. In addition, the study by Suzuki et al. (26) found that the elderly were also more prone to lumbar muscle strain and paraspinal muscle fat infiltration. All these factors are related to long-term chronic low back pain, and postoperative recurrence and low back pain will cause postoperative complications.

Occupation is also an important risk factor, and the results of this study suggest that people who engage in heavy physical activity or occupations that require prolonged sedentary standing may have poorer postoperative clinical outcomes than other patients. Salo et al. (27). conducted a prospective cohort study, which included 1022 patients and assessed the degree of intervertebral disc degeneration by measuring the Pfirrmann grade of lumbar intervertebral discs on MRI, and then the relationship between occupational physical load and intervertebral disc degeneration was assessed. The results of the study showed that higher occupational physical load was associated with more severe intervertebral disc degeneration, and the results of the study by Kong et al. were consistent with our findings (28).

The results of this study suggest that even if the symptoms of patients improve after surgery, attention should be paid to the health education of patients, and even if the continued influence of occupational factors cannot be completely avoided, appropriate rest should be taken after the lumbar spine is overloaded.

In this study, the proportion of patients with osteoporosis in group B was significantly higher than that in group A, and the difference was statistically significant, indicating that osteoporosis was also an important factor affecting the improvement rate of the lumbar spine JOA score after TMD. The study by Miyagi et al. (29) showed that osteoporosis had a strong correlation with the lumbar spine JOA score, and high bone turnover in patients with osteoporosis was a risk factor for gait disturbance in JOA. Although the mechanism is unclear, osteoporosis treatment may improve the patient's gait ability. The study by Hikata et al. (30) also showed that osteoporosis was closely related to the satisfaction of patients after LDH, which may be related to the fact that osteoporosis often leads to low back pain and gait disturbance in patients. In previous studies, people paid more attention to the study of the relationship between the fusion rate and osteoporosis after lumbar fusion and internal fixation, while the study on the correlation between the postoperative curative effect and osteoporosis after minimally invasive lumbar spine surgery without fusion was rare, but it should still be given adequate attention.

Pfirrmann grading (13) is a commonly used grading method for assessing the degree of lumbar intervertebral disc degeneration. In this study, the Pfirrmann grade of all patients was grade 2 or above, indicating that lumbar intervertebral disc degeneration was the pathogenesis of LDH. The results of the nomogram in this study showed that the higher the Pfirrmann grade, the worse the clinical efficacy after TMD. This may be because patients with severe disc degeneration are more likely to have chronic low back pain, which is also consistent with the results presented by Smith and Lambrechts et al. (31). The results of studies are consistent, but the mechanism is still unclear and needs further study (32). It is worth noting that more severe degeneration of the intervertebral disc does not indicate a higher probability of postoperative recurrence. The study by Belykh et al. (33, 34) showed that patients with Pfirrmann grade 3 had the highest probability of postoperative recurrence of lumbar intervertebral disc herniation. As the nucleus pulposus and annulus fibrosus have basically disappeared in patients with Pfirrmann grades 4 or 5, they are not prone to recurrence.

The ODI rating scale is a widely used scale for assessing low back pain dysfunction (14). The results of this study showed that the higher the preoperative ODI score, the worse the clinical efficacy after TMD. The value of ODI score in assessing the degree of dysfunction has been proved in many studies. A high preoperative ODI score means that there is severe functional disability before surgery, and many studies have also proved that preoperative ODI score can accurately predict postoperative lumbar disc surgery (35). The study by Puolakka et al. (36) found that ODI score was an important predictor of work ability after lumbar disc surgery, and the higher the preoperative ODI score, the greater the loss of postoperative work ability. Therefore, the preoperative ODI score is also an important factor affecting the postoperative efficacy of TMD.

Through scientific follow-up, data collection, and statistical analysis, a clinical prognosis model with good performance was constructed in this study, but there are still several limitations. (1) This study was a retrospective observational study with a low level of evidence; (2) This study only used the internal data set to verify the model internally, and lacked external data to verify the model externally; (3) For other clinical outcomes with low probability of occurrence, an inadequate sample size resulted in insufficient statistical power. Therefore, it is impossible to perform an effective statistical analysis of the clinical outcomes with small probability, such as postoperative recurrence and intraoperative complications, and the study also lacks the analysis and evaluation of other clinical outcomes, such as quality of life and satisfaction. The prognosis after 1 year was followed up, but the early and late postoperative follow-up data were lacking, and the statistical analysis of the early and late prognosis was not carried out. Therefore, we need to conduct multicenter prospective studies with large sample sizes to obtain data sets with higher levels of evidence to better construct, optimize, validate, and evaluate models to predict this prognosis.

## 5. Conclusion

This study successfully developed a clinical prediction model and a web calculator with good performance that can predict the effect of TMD for LDH, and it is easy to perform and can be widely used in daily clinical work.

## Data Availability

The raw data supporting the conclusions of this article will be made available by the authors, without undue reservation.
